# Efficacy of denosumab for restoring normal bone mineral density in women receiving adjuvant aromatase inhibitors for early breast cancer

**DOI:** 10.1097/MD.0000000000016770

**Published:** 2019-08-09

**Authors:** Koichi Sakaguchi, Hisako Ono, Katsuhiko Nakatsukasa, Takashi Ishikawa, Yoshie Hasegawa, Masato Takahashi, Naoki Niikura, Kei Koizumi, Teruhisa Sakurai, Hideo Shigematsu, Shunji Takahashi, Shinichiro Taira, Masato Suzuki, Kazutaka Narui, Daishu Miura, Kimito Yamada, Mana Yoshimura, Hisashi Shioya, Eiichi Konishi, Yokota Isao, Kojiro Imai, Kei Fujikawa, Tetsuya Taguchi

**Affiliations:** a Division of Endocrine and Breast Surgery, Kyoto Prefectural University of Medicine, Kawaramachi-hirokoji, Kamigyo-ku, Kyoto; b Drug Discovery Center, Graduate School of Medicine; c Department of Surgical Pathology, Kyoto Prefectural University of Medicine; d Department for Medical Innovation and Translational Medical Science; e Kyoto Prefectural University of Medicine Graduate School of Medical Science; f Department of Biostatistics, Kyoto Prefectural University of Medicine Graduate School of Medical Science; g Department of Breast Oncology; h Department of Radiology, Tokyo Medical University; i Department of Breast Surgical Oncology, Hirosaki Municipal Hospital; j Department of Breast Surgery, Hokkaido Cancer Center; k Department of Breast and Endocrine Surgery, Tokai University School of Medicine; l Department of Breast Surgery, Hamamatsu University School of Medicine; m Department of Surgery, Wakayama Medical University Kihoku Hospital; n Department of Breast Surgery, National Hospital Organization Kure Medical Center and Chugoku Cancer Center; o Department of Medical Oncology, Cancer Institute Hospital of Japanese Foundation for Cancer Research; p Department of Breast Surgery, National Hospital Organization Chiba Medical Center, Yokohama City University Medical Center; q Department of Breast and Thyroid Surgery, Yokohama City University Medical Center; r Akasaka Miura Clinic, Tokyo; s Department of Surgery, The Jikei University School of Medicine; t Department of Biostatistics, Graduate School of Medicine, Hokkaido University, Nakatsukasa Adachi Clinic, Japan.

**Keywords:** aromatase inhibitor, bone health, bone mineral density, hormone-sensitive breast cancer, postmenopausal

## Abstract

**Background:**

Osteoporosis is a major side effect of aromatase inhibitors (AIs), which are greatly effective in the treatment of breast cancer. However, there are no satisfactory measures against osteoporosis. In this multicenter, randomized, comparative study, we evaluate the efficacy of denosumab for preventing loss of bone mineral density (BMD) induced by adjuvant therapy with AI s in breast cancer patients with normal BMD.

**Patients and methods:**

The bone loss-suppressing effect of denosumab will be comparatively evaluated in postmenopausal patients scheduled to receive letrozole or anastrozole as a postoperative endocrine therapy for stage I–IIIA hormone-sensitive breast cancer and a control group. Patients will be administered letrozole 2.5 mg or anastrozole 1 mg once a day, and the treatment will be continued for 5 years unless recurrence, secondary cancer, or unacceptable toxicity develops. Patients in the denosumab group will receive a subcutaneous injection of 60 mg of denosumab every 6 months. The primary endpoint is the rate of change in the lumbar spine (L1–L4) BMD, as determined by dual-energy X-ray absorptiometry (DXA), 12 months after the start of the injection. The secondary endpoints were

(1) rate of change in the lumbar spine (L1–L4) BMD after 2, 3, 4, and 5 years;
(2) rate of change in the femoral neck BMD;
(3) rate of change in radius BMD (determined using an ultrasonic bone densitometer);
(4) changes in calcium and bone metabolism markers (TRAP 5b, bone alkaline phosphatase, and pentosidine);
(5) development of pathological fracture within 3 years;
(6) disease-free survival;
(7) overall survival (OS);
(8) adverse events; and
(9) quality of life.

**Ethics and dissemination:**

The protocol was approved by the institutional review boards of Kyoto Prefectural University of Medicine and all the participating faculties. Written informed consent was obtained from all patients before registration, in accordance with the Declaration of Helsinki. Results of the study will be disseminated via publications in peer-reviewed journals.

**Trial registration:**

Clinical Trials.gov Identifier: NCT03324932, Japan Registry of Clinical Trial (jRCT): CRB5180001.

## 1 Introduction

The incidence of breast cancer is increasing annually, and in approximately 70% or more cases, the cancer is hormone-positive. Postoperative administration of aromatase inhibitors (AIs) is essential in many postmenopausal patients with hormone-positive breast cancer. With regard to adjuvant therapy for hormone-positive breast cancer patients, the standard therapies are tamoxifen for 5 years in premenopausal women and AIs for 5 years in postmenopausal women. However, in recent years, much data have been reported on the usefulness of long-term adjuvant endocrine therapy for patients with hormone-positive breast cancer.^[1–3]^ Long-term hormonal therapy for more than 5 years contributes to the reduction of recurrence rate, prevention of contralateral breast cancer, and thus an improvement in survival rate; however, the disadvantages cannot be ignored. In the case of long-term administration of an AI, in particular, a decrease in bone density is an important issue that cannot be avoided.

Prolonged AI administration in postmenopausal patients with breast cancer reduces bone mass because of estrogen depletion. Therefore, for bone loss prevention, an intermittent subcutaneous injection of denosumab is administered every 6 months in combination with letrozole and anastrozole to patients with early-stage breast cancer patients scheduled to undergo postoperative endocrine therapy.

Letrozole and anastrozole are both reversible AIs. Letrozole (Femara) is a type II reversible AI synthesized by Novartis Pharma (formerly Ciba-Geigy), Switzerland. It shows more potent aromatase inhibitory activity and estrogen suppression compared to those shown by anastrozole (Arimidex), another type II reversible AI. Denosumab is a humanized IgG2 monoclonal antibody preparation that targets receptor activator of NF-κB ligand (RANKL). RANKL is an essential mediator for the formation, function, and survival of osteoclasts binding to RANK on osteoclasts and surface of osteoclast precursor cells. This drug specifically inhibits RANKL and inhibits bone resorption by osteoclasts. Pralia (denosumab) is a novel agent for osteoporosis treatment; it specifically inhibits RANKL, which is an essential mediator for bone resorption. The agent is subcutaneously administered once every 6 months. In a Japanese Phase III clinical trial (the DIRECT study) conducted in patients with osteoporosis, the cumulative incidence of vertebral fracture suppression associated with Pralia was significantly different from that associated with placebo. In randomized controlled trials with placebo among postmenopausal patients with hormone-positive breast cancer who received AIs, Pralia significantly increased bone density.

The target of this study is postmenopausal Japanese women with hormone receptor-positive resected breast cancer of clinical stage I–IIIA who are scheduled to be administered letrozole 2.5 mg or anastrozole 1 mg once a day as a postoperative endocrine therapy. The study design is aimed at comparing the results of the denosumab group administered with denosumab every 6 months with those obtained for the control group in addition to comparing the results with clinical trials conducted overseas.

## 2 Endpoints

### 2.1 Primary

The primary endpoint is the rate of change in the lumbar spine (L1–L4) BMD 12 months after the start of the study. The change rate is expressed as a percentage of a value obtained by subtracting 1 from the BMD/baseline BMD value after 12 months.

### 2.2 Secondary

(1) Rate of change in the lumbar spine (L1–L4) BMD: after 2, 3, 4, and 5 years
(2) Rate of change in the femoral neck BMD: 12 months, and 2, 3, 4, and 5 years
(3) Rate of change in the radius BMD (determined using an ultrasonic bone densitometer): after 2 weeks, 4 weeks, and every 4 weeks thereafter (2 years from registration)
(4) Rate of change in calcium and bone metabolism markers (TRAP 5b, bone-type alkaline phosphatase (BSAP), and blood pentosidine)
(5) Incidence rate of pathological fracture within 3 years
(6) Disease-free survival (DFS)
(7) OS
(8) Adverse events (such as hypocalcemia and osteonecrosis of the jaw bone) (type/rate of occurrence)
(9) Quality of life (QoL): Euro-QoL Japanese version (EQ-5D-5L).

## 3 Methods and analysis

### 3.1 Study design

This study is a multicenter, randomized, comparative study.

### 3.2 Study setting

Seventeen hospitals agreed to participate in this study. The protocol was approved by the central review board of Kyoto Prefectural University of Medicine. Written informed consent was obtained from all patients before registration, in accordance with the Declaration of Helsinki. Patients are registered in this study after an independent review by The Center for Quality Assurance in Research and Development (CQARD), Kyoto, where potential subjects are screened against the inclusion and exclusion criteria. At least annual independent monitoring is planned, in accordance with the Japanese clinical trial guideline.

**Figure 1:**
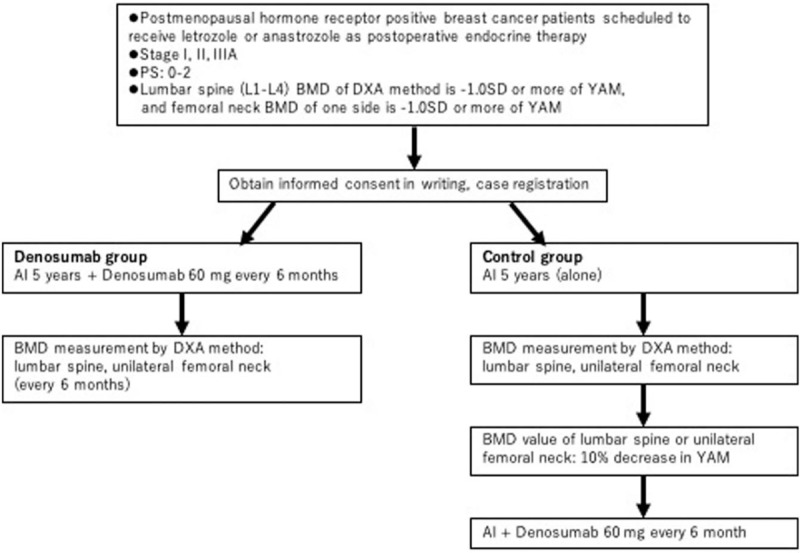
Trial Regimens. Patients are administered letrozole 2.5 mg or anastrozole 1 mg once a day, continued for 5 years. For the denosumab group, patients are administered denosumab subcutaneously at a dose of 60 mg every 6 months. The Primary endpoint is the rate of change of the lumbar spine (L1-L4) BMD by the DXA method 12 months after the start of the injection.

Dosing is as per the study design. Patients in the control group will receive letrozole 2.5 mg or anastrozole 1 mg once daily after breakfast as a postoperative endocrine treatment for 5 years. In the denosumab group, in addition to oral administration of letrozole 2.5 mg or anastrozole 1 mg, denosumab 60 mg is administered subcutaneously every 6 months (Fig. 1). If the lumbar spine (L1–L4) or unilateral femoral neck BMD value decreases by 10% or more in the control group during treatment, a subcutaneous injection of denosumab 60 mg will be administered every 6 months while oral treatment continues. During the study, administration of other endocrine therapy agents, antineoplastic agents, drugs affecting bone metabolism (calcitonin, raloxifene, PTH preparation, vitamin K preparation, strontium preparation, active vitamin D preparation, ipriflavone preparation, systemic corticosteroids) and bisphosphonate are prohibited.

If it is determined that discontinuation of hormonal agents is appropriate in the denosumab group and control group, this study is considered as discontinuation, and the discontinuation date, reason for discontinuation, and outcome at discontinuation are described in the case report form.

### 3.3 Study timeline

Study period: January 2017 to December 2026.

Case registration period: January 2017 to December 2021.

### 3.4 Patients

The inclusion criteria are as follows:

(1) Invasive breast cancer patients over 20 years of age who meet the following requirements
  - Patients diagnosed with stage I, II, or IIIA cancer based on pathological testing
  - Patients who received an appropriate operation such as mastectomy and breast conservation.
(2) Patients for whom immunohistochemistry showed positivity for at least 1 of estrogen receptor (ER) and progesterone receptor (PgR)
(3) Women who meet the following menopause criteria
  - Non-menstruating women aged 55 years or more
  - Age less than 55 years, amenorrhea for more than 12 months, determined to be menopause based on follicle-stimulating hormone (FSH) and estradiol levels
  - Patients who had undergone bilateral ovariectomy.
(4) Lumbar spine (L1–L4) BMD before the start of the test, as determined by dual-energy X-ray absorptiometry (DXA), is more than or equal to -1.0 SD of female young adult mean (YAM)
(5) Patients with Eastern Cooperative Oncology Group (ECOG) performance status (PS) of 0-2
(6) Patients with appropriate organ function (within 4 weeks prior to case registration)
  - Leukocyte count 3000/mm 3 or neutrophil count of 1500 1500/mm 3
  - Aspartate aminotransferase (AST) and alanine aminotransferase (ALT) levels ≤1.5 times the upper limit of the facility standard value
  - Serum creatinine level of ≤1.5 times the upper limit of the facility standard value.
(7) Case registration will be carried out by the following time point
  - Within 12 weeks after completion of surgery or postoperative chemotherapy.
(8) Patients who have taken for more than 4 weeks after discontinuing use of bisphosphonates (oral agents), estrogen preparations, raloxifene, calcitonin preparations, vitamin K preparations, active vitamin D preparations, and ipriflavone preparations that are known to affect bones
(9) Patients who provide written informed consent to participate in the study.

The exclusion criteria are as follows:

(1) Patients with distant metastasis
(2) Patients with bilateral breast cancer
(3) Patients who started postoperative hormonal therapy before obtaining consent
(4) Patients who received endocrine therapy within 52 weeks before obtaining consent
(5) Patients who have had intravenous administration of bisphosphonates within 52 weeks before obtaining consent
(6) Patients with the following diseases that interfere with DXA measurement:
(7) Severe scoliosis, immobility, lumbar spine hyperostosis or osteosclerosis, abdominal aortic calcification, or spinal disease
(8) Patients with a history of malignant tumors other than breast cancer within 260 weeks before obtaining consent
(9) Patients with a dental disease such as a dental or jaw bone infection or dental trauma at 6 weeks or more after obtaining consent
(10) Patients scheduled to have teeth or jaw surgery within 6 weeks after obtaining consent (such as an extraction or implant)
(11) Any other patients who are regarded as unsuitable for this study by the investigators.

### 3.5 Rationale for the setting of the number of enrolled patients

In the clinical trial (ABCSG-18 trial) that investigated the combined effect of denosumab on bone loss due to letrozole or anastrozole as a postoperative endocrine therapy for postmenopausal breast cancer patients, in the denosumab group, the lumbar spine BMD at 12 months (5.75%; standard deviation: 8%) was significantly higher than that in the placebo administration group (*P* <.0001).

Based on this result, for postoperative endocrine therapy with letrozole or anastrozole for postmenopausal breast cancer patients in Japan, it is assumed that the difference in the lumbar spine BMD after 12 months is 4% between the denosumab group and the placebo group. In order to calculate with a significance level of 2.5% and a power of 80%, 64 cases per group are required. Given that 20% of patients dropped out after 12 months, the number of randomized patients required is 80 per group.

### 3.6 Statistical methods

Study data will be managed at Kyoto Prefectural University of Medicine Research and Development and Quality Management Improvement Integration Center. The intention-to-treat population will be targeted at cases randomly assigned to either the denosumab or control group. The efficacy analysis will be conducted in the full analysis set excluding cases out of the eligibility criteria, cases not receiving the study drug (AI or denosumab), cases not having any efficacy data after randomization, from the cases randomly assigned. A safety assessment will be conducted in a safety analysis population for all patients who were randomized and given the study drug (AI or denosumab) at least once. The primary endpoint is the rate of change in the lumbar spine (L1-L4) BMD after 12 months from baseline, as determined by DXA. We will use analysis of covariance including treatment group, BMD values at baseline, the existing of neo-adjuvant or adjuvant chemotherapy, stage and age. As a sensitivity analysis of missing BMD at 12 months, the last observation carried forward method will be used. Selection model or pattern-mixture model may be applied depending on the occurrence of missing. One-sided significance level was set to 0.025.

### 3.7 Ethics

The trial received ethical approval from the Ethics Committee of Kyoto Prefectural University of Medicine, Kyoto, Japan (CRB5180001, last edition 27/3/2018). The trial is subject to the supervision and management of the ethics committee. This study is registered with Clinical Trials.gov (Identifier NCT03324932) and Japan Registry of Clinical Trial (jRCT; CRB5180001).

## 4 Discussion

Estrogen acts on both osteoblasts and osteoclasts to suppress bone remodeling (bone turnover) and plays an important role in bone mass maintenance among healthy premenopausal adult females.^[4]^ However, in postmenopausal women, estrogen secretion from the ovaries stop; therefore, generally, the bone remodeling rate increases and the bone mass decreases.

Postoperative endocrine therapy with AIs for early breast cancer significantly improved DFS compared to the DFS achieved with tamoxifen, which was a conventional standard therapy in several large-scale clinical trials such as ATAC,^[5]^ BIG 1–98,^[6]^ and IES.^[7]^ However, postoperative endocrine therapy with AIs results in an almost complete loss of estrogen in postmenopausal women, and a decrease in BMD is observed as bone turnover rapidly and continually increases.^[8]^ In addition, clinical trials revealed that the fracture rate in patients who received AI increased compared to that in patients who received tamoxifen.^[9]^

In a Phase III clinical trial (FREEDOM study) conducted overseas, postmenopausal osteoporosis patients treated with denosumab 60 mg showed significantly increased BMD of the lumbar spine and femur compared to that in the placebo group, indicating denosumab's excellent efficacy.^[10]^ Denosumab also was shown to increase BMD continuously for 8 years, suppressing vertebral body fractures and non-vertebral fractures. In a domestic Phase III trial (the DIRECT study), denosumab significantly increased the BMD of the lumbar vertebrae (L1 to L4), femoral neck, and radial distal end 1/3 at 12 months and 24 months after administration compared to that in the placebo group and suppressed the development of vertebral fractures, indicating efficacy in a Japanese population.^[11]^

In the clinical trial ABCSG-18 using denosumab for the purpose of inhibiting bone loss associated with postoperative endocrine therapy by AIs of early-stage breast cancer patients conducted, the denosumab group showed improved BMD of the lumbar spine and femur compared to the placebo group. Which means that denosumab suppresses the decrease in bone mass accompanying the AI.

A clinical trial (ABCSG-18) was conducted to investigate the combined effect of denosumab on bone loss due to AI administration as postoperative endocrine therapy for postmenopausal breast cancer patients. In this trial, in the denosumab-administered group, the lumbar spine BMD value at 12 months, which was the primary endpoint, was significantly higher (5.75%) than that in the placebo group (*P* <.0001) and the fracture rate was significantly lower during the 36-month observation period (*P* <.0001). In addition, the 5-year DFS rate tended to be higher in the administration group than in the placebo group. The results of the analysis of OS rates are currently awaited.^[12]^ If results similar to those of the ABCSG-18 trial are obtained in our study, it would confirm not only a preventive effect against bone mass reduction by AI in a Japanese population but also a simultaneous recurrence-suppressive effect, and the significance as a postoperative adjuvant therapy is high.

We have reported the efficacy of denosumab in patients with low BMD undergoing adjuvant AI therapy for Japanese breast cancer. At 12 months, the lumbar spine BMD increased by 4.7%. Patients who received AIs before denosumab (n = 70) showed a 4.7% increase in BMD, and those who received denosumab at the start of AI therapy (n = 30) showed a 4.5% increase in BMD (*P* = .8385). Additionally, a 2.4% and 1.4% increase was observed in the BMD of the right and left femoral neck, respectively.^[13]^ For more meaningful research, analysis of the abovementioned results and the results for patients with normal bone density should be integrated.

Consider the problem from another point of view, the most frequent organ as a metastatic organ of breast cancer is bone. The molecular biological mechanism of bone metastasis of breast cancer has not yet been fully elucidated. It is clear that the expression of RANKL and its receptor RANK play important roles in bone remodeling (bone resorption and bone formation) as well as in breast cancer, prostate cancer, renal cancer, and malignant melanoma. There are reports that expression of RANKL/RANK induces bone metastasis. In breast cancer, expression of RANK is visualized using microarray and immunostaining, and it is reported that high RANK expression leads to a poor prognosis compared to that associated with low RANK expression. RANK expression has been suggested to be used as a prognostic factor.^[14]^

Thus, the relationship between breast cancer and bone is very close, and reduction of the effect on bone while treating breast cancer can be a very important issue. In this regard, we believe that the results of our study would be important in evaluating the usefulness of denosumab in the treatment of Japanese breast cancer patients. We are planning to conduct an additional study using pathology samples from this study. We retrospectively examined the expression of RANK, RANKL, OPG, and so on. in relapsed breast cancer cases and recurrence-free cases, and clarify the relationship between RANK, RANKL, OPG expression, and prognosis in Japanese breast cancer.

## Acknowledgments

We thank the patients and their families and all the investigators involved in this study.

## Author contributions

Conceptualization and design: Tetsuya Taguchi, Katsuhiko Nakatsukasa, Hisako Ono, Takashi Ishikawa, Shunji Takahashi, Isao Yokota, Kojiro Imai.

Investigation: Koichi Sakaguchi, Hisako Ono, Katsuhiko Nakatsukasa, Takashi Ishikawa, Yoshie Hasegawa, Masato Takahashi, Naoki Niikura, Kei Koizumi, Teruhisa Sakurai, Hideo Shigematsu, Shunji Takahashi, Shinichiro Taira, Masato Suzuki, Kazutaka Narui, Daishu Miura, Kimito Yamada, Mana Yoshimura, Hisashi Shioya, Eiichi Konishi, Tetsuya Taguchi

Data management: Kei Fujikawa

Formal analysis: Isao Yokota

Manuscript preparation: Koichi Sakaguchi

Manuscript editing: Tetsuya Taguchi

Project administration: Koichi Sakaguchi, Hisako Ono, Katsuhiko Nakatsukasa, Kojiro Imai

Supervision: Tetsuya Taguchi.

**Conceptualization:** Hisako Ono, Katsuhiko Nakatsukasa, Takashi Ishikawa, Shunji Takahashi, Yokota Isao, Kojiro Imai, Tetsuya Taguchi.

**Data curation:** Kei Fujikawa.

**Formal analysis:** Yokota Isao.

**Investigation:** Koichi Sakaguchi, Hisako Ono, Katsuhiko Nakatsukasa, Takashi Ishikawa, Yoshie Hasegawa, Masato Takahashi, Naoki Niikura, Kei Koizumi, Teruhisa Sakurai, Hideo Shigematsu, Shinichiro Taira, Masato Suzuki, Kazutaka Narui, Daishu Miura, Kimito Yamada, Mana Yoshimura, Hisashi Shioya, Eiichi Konishi, Tetsuya Taguchi.

**Project administration:** Koichi Sakaguchi, Hisako Ono, Katsuhiko Nakatsukasa, Kojiro Imai.

**Supervision:** Tetsuya Taguchi.

**Writing –oiginal daft:** Koichi Sakaguchi.

**Writing – rview & eiting:** Tetsuya Taguchi.

Koichi Sakaguchi orcid: 0000-0003-1412-0604.
